# Recommendations for 46,XY Disorders/Differences of Sex Development Across Two Decades: Insights from North American Pediatric Endocrinologists and Urologists

**DOI:** 10.1007/s10508-024-02942-1

**Published:** 2024-07-22

**Authors:** Behzad Sorouri Khorashad, Melissa Gardner, Peter A. Lee, Barry A. Kogan, David E. Sandberg

**Affiliations:** 1 Department of Pediatrics, Susan B. Meister Child Health Evaluation and Research Center, Michigan Medicine, University of Michigan Medical School, 2800 Plymouth Road, Ann Arbor, MI 48109-2600 USA; 2 Department of Pediatrics, Penn State College of Medicine, Hershey, PA USA; 3 Department of Urology, Albany Medical College, Albany, NY USA; 4 Division of Pediatric Psychology, Department of Pediatrics, University of Michigan Medical School, Ann Arbor, MI USA

**Keywords:** Partial androgen insensitivity syndrome, Micropenis, Penile ablation, Disorders of sex development, Gender assignment, Optimal gender policy

## Abstract

Clinical decision-making for individuals with 46,XY disorders/differences of sex development (DSD) remains unsettled and controversial. The North American DSD Clinician Survey examines the recommendations of a large group of clinical specialists over the last two decades. Active members of the (Lawson Wilkins) Pediatric Endocrine Society and the Societies for Pediatric Urology were invited to respond to a web-based survey at three different timepoints: 2003–2004 (T1), 2010–2011 (T2), and 2019–2020 (T3). Data from 429 participants in T1, 435 in T2, and 264 in T3 were included in this study. The participants were presented with three XY newborn clinical case scenarios—micropenis, partial androgen insensitivity syndrome, and iatrogenic penile ablation—and asked for clinical management recommendations. The main outcomes assessed included the recommended gender of rearing, surgical decision-maker (parent or patient), timing of genital surgery, and age at which to disclose medical details and surgical history to the patient. For all scenarios, the overwhelming majority recommended rearing as male, including a significant increase across timepoints in those recommending a male gender of rearing for the infant with penile ablation. The proportions recommending female gender of rearing declined significantly across timepoints. In general, most recommended parents (in consultation with the physician) serve as surgical decision-makers, but these proportions declined significantly across timepoints. Recommendations on the timing of surgery varied based on the patient’s gender and type of surgery. There has been a shift in recommendations away from the “optimal gender policy” regarding gender of rearing and surgical interventions for patients with XY DSD.

**Supplementary Information:**

The online version contains supplementary material available at 10.1007/s10508-024-02942-1.

## Introduction

Disorders/differences of sex development (DSD) is an umbrella term for a group of congenital conditions in which the development of chromosomal, gonadal, or anatomical sex is atypical and can be classified into three categories based on the sex chromosome arrangements: (1) sex chromosome DSD; (2) 46,XX DSD; and (3) 46,XY DSD (Lee et al., 2006). Depending on the underlying pathophysiology, 46,XY DSD can be subclassified into (1) disorders of testis development; (2) disorders of androgen synthesis; (3) disorders of androgen action; and (4) other conditions affecting sex development (Chan et al., 2020). 46,XY DSD can present with a wide spectrum of phenotypes, ranging from female-typical external genitalia (as observed in complete androgen insensitivity syndrome [CAIS] or complete testicular dysgenesis) to clitoromegaly or micropenis (as seen in partial androgen insensitivity syndrome [PAIS], 17β-hydroxysteroid dehydrogenase type 3 deficiency [17βHSD3], or 5α-reductase type 2 deficiency [5αRD2]), and anatomic anomalies such as cloacal exstrophy (Reiner & Gearhart, 2004). Clinical management in patients with 46,XY DSD has been and remains controversial. Moreover, although not classified as a DSD, the management of healthy male newborns whose penis was iatrogenically ablated–most commonly during a circumcision–has been equally controversial and historically discussed alongside 46,XY DSD conditions (Meyer-Bahlburg, 2005).

One of the most significant aspects in the clinical management of patients with 46,XY DSD is the decision regarding gender of rearing. Deciding whether to rear such a newborn as a boy, girl, or non-binary, is expected to have consequences for the long-term outcomes of the patient’s mental health and quality of life across the lifespan. Threats to positive outcomes include the development of a gender identity discordant with the gender of rearing, experiencing one’s sexual life as unsatisfactory, or developing a negative body and self-image (Zucker, 1999).

Beginning in the 1950s, Money et al. (1957) conducted studies on the psychosexual development of children born with markedly atypical genitalia. Their findings, consistent with previous conceptualizations (Bentley, 1945; Ellis, 1945; Money, 1955), supported the conclusion that gender of rearing was a more reliable predictor of the person’s gender identity than either chromosomal or gonadal sex. Replacing efforts at identifying the person’s “true sex” through examination of the gonads, what came to be referred to as “optimal gender policy” (Meyer-Bahlburg, 1998), considered multiple aspects of psychosexual outcome, most prominently the potential for penetrative intercourse. To achieve stability of gender identity and positive psychological adaptation, the optimal gender policy assumed the necessity of genital appearance and function that matched the gender of rearing (Stein et al., 2003).^1^ However, a major barrier to rearing 46,XY newborns with marked genital atypia as boys has been the limitations in surgically creating a functional penis. As a consequence, a female gender of rearing accompanied by surgical creation of a vagina was considered likely to be more successful (Lee & Houk, 2013).

The assumption that gender identity follows gender of rearing subsequently came into question. In several examples of 46,XY DSD, including 5αRD2 and 17βHSD3 (Imperato-McGinley et al. (1974), cloacal exstrophy (Reiner et al., 2004), and penile agenesis (Meyer-Bahlburg (2005), those reared as girls were found to be at increased likelihood of later patient-initiated gender reassignment compared with those originally reared as boys. In the case of patients with DSD attributable to PAIS, only a minority (~ 10%) self-initiated gender reassignment–whether originally reared as boys or as girls. Yet this incidence of gender change was also substantially higher than in the general population (Mazur, 2005). A review of the stability of gender identity in individuals born with a micropenis (i.e., stretched penile length ≤  − 2.5 SD for age and stage of puberty) revealed that the vast majority (~ 90%) had been reared as boys. Whether reared as a boy or as a girl, none changed their gender (Mazur, 2005). However, in one study of adults born with a micropenis and reared as girls (5 of 18; 28%), all expressed satisfaction with their gender of rearing, despite 4 out of these 5 reporting having previously questioned their gender assignment (Wisniewski et al., 2001). Extending the principles of the optimal gender policy to a biologically typical male whose penis was accidentally totally ablated during a circumcision accident, John Money recommended that the child be reared as a girl and receive feminizing surgery (Money, 1975). However, accounts of this individual’s psychosexual development during adolescence and beyond indicated a self-initiated gender change back to male (Diamond, 1982; Diamond & Sigmundson, 1997). Although not categorized as a DSD, this single penile ablation case has had a magnified influence on the opinions of clinicians (Yang et al., 2010), and the general public (Colapinto, 1997, 2000), despite a published report of a similar case of traumatic amputation of the penis in infancy with a different outcome (Bradley et al., 1998).

The 2006 Consensus Statement on Management of Intersex Disorders (Lee et al., 2006), which first introduced the umbrella term “disorders of sex development” and their categorization according to sex chromosomes, recommended that patients with micropenis be reared as boys. The 2016 update of the consensus statement reinforced this recommendation (Lee et al., 2006). Neither of the two statements were prescriptive regarding the gender of rearing in PAIS. Nonetheless, the 2016 consensus update suggested that gender of rearing “should be based upon a demonstrable response with phallic growth to testosterone therapy and genetic assessment if a causative variant of the gene is found, while female assignment must be considered for those without evidence of androgen effects” (Lee et al., 2006, p. 169). Because traumatic amputation of the penis in the newborn is not a DSD, the 2006 consensus statement and its 2016 update are silent regarding recommendations for gender of rearing. However, it can be inferred from the recommendation to rear as boys newborns with 46,XY DSD due to cloacal exstrophy or penile agenesis due to presumed typical-male prenatal androgen exposure, that male gender of rearing would be recommended in the case of an iatrogenically-ablated penis.

With regard to elective surgery to reconstruct the genitalia and its timing, the 2006 consensus statement emphasized “functional outcome rather than a strictly cosmetic appearance” (Lee et al., 2006, p. e491) and partially relied on a 1996 guidance from the Section of Urology of the American Academy of Pediatrics to justify initiating masculinizing surgery between 6 to 12 months of life (American Academy of Pediatrics & Section on Urology, 1996). The 2006 statement also recognized that “Feminizing genitoplasty as opposed to masculinizing genitoplasty requires less surgery to achieve an acceptable outcome and results in fewer urologic difficulties” (Lee et al., 2006). By the time of the 2016 consensus update, agreement regarding the indications for or the timing of elective surgery in DSD remained out of reach (Lee et al., 2006). In stark contrast to the apparent commitment of the medical community to innovate surgical techniques, the intersex advocacy movement, which emerged in the early 1990’s, was clear in its calls for a moratorium on early non-emergent genital surgery (Chase, 2003; Diamond, 1999; Sandberg & Vilain, 2022; Sudai, 2018).

The rise of intersex activism (Chase, 2003) and a shift away from medical paternalism toward the model of patient-centered care (Institute of Medicine Committee on Quality of Health Care in America, 2001) contributed to increased openness in educating patients. The 2006 consensus statement, and related white papers that followed (Cools et al., 2018; Lee et al., 2006, 2016; Wisniewski et al., 2001) uniformly called for openness in disclosing to the patient facts about their diagnosis, its implications, and history of medical and surgical care, even though these authoritative reports were short on specifics regarding optimal timing and methods.

The aim of this study, as part of a larger project, was to explore how experts whose specialties are central in DSD care (i.e., pediatric endocrinology and urology), recommend managing various aspects of the clinical care of children born with 46,XY DSD. Additional aims included assessing whether these recommendations aligned with authoritative and contemporaneous white papers, and how (if at all) these recommendations have changed over the last two decades. We hypothesized that shifts away from the optimal gender policy would be associated with DSD clinical specialists recommending the rearing of 46,XY newborns with marked genital atypia as boys. We also predicted an increasing proportion, across survey timepoints, would recommend including the patient in surgical decision-making (associated with a parallel delay in the timing of surgery), and a progressively earlier disclosure of the medical and surgical history to the patient. This study also investigated potential differences in the clinical recommendations between specialty and personal demographics of survey participants.

## Method

### Participants

Active members of the (Lawson Wilkins) Pediatric Endocrine Society (PES) and the Societies for Pediatric Urology (SPU), as listed in the respective membership directories, were targeted for participation at three timepoints: 2003–2004 (T1), 2010–2011 (T2), and 2020 (T3).^2^ The inclusion criteria were: (1) active membership of PES or SPU; (2) working within North America (US, Canada, Mexico); (3) specializing in endocrinology or urology; and (4) caring for patients with DSD.^3^ The total number of participants completing survey items pertaining to management of 46,XY cases were n = 429 (PES: 297; SPU: 132) at T1; n = 435 (PES: 319; SPU: 116) at T2; and n = 264 (PES: 114; SPU: 150) at T3. Citing concerns over burden on its members, PES did not endorse member participation at T3. Consequently, only those pediatric endocrinologists who had previously been invited to participate at T1 or T2 were solicited. A PES member directory was reviewed to remove names of those who were no longer listed as members (Table 1).

**Table 1:** Participant demographic and professional characteristics at each survey timepoint

		2003 (n = 429)		2010 (n = 435)		2020 (n = 264)	
		n	%	n	%	n	%
Specialty	PES	297	69.2	319	73.3	114	43.2
	SPU	132	30.8	116	26.7	150	56.8
Gender	Male	306	71.3	268	61.6	184	69.7
	Female	123	28.7	167	38.4	80	30.3
Practice setting	Medical school or hospital	290	67.6	319	73.3	205	77.7
	Other	139	32.4	116	26.7	59	22.3
Practice country	United States	404	94.2	412	94.7	249	94.3
	Canada	25	5.8	23	5.3	15	5.7
		Mean	SD	Mean	SD	Mean	SD
Birth year		1951.1	9	1958.9	10	1963.7	10
Clinical experience (DSD cases/career)		50	100	43	168	50	341

*PES* Pediatric Endocrine Society, *SPU* Societies for Pediatric Urology, *DSD* Differences/disorders of sex development, *SD* Standard deviation

At all three timepoints, the majority of survey participants were male and this varied little over the three timepoints (Wald *χ*^2^(2) = 14.66, *p* < 0.001; male percentage: T1: 71.3%, T2: 61.6%, T3: 69.7%, Table 2). At T1 and T2, most of participants were PES members, but at T3 the majority were SPU members (Wald *χ*^2^(2) = 69.39, *p* < 0.001; PES percentage T1: 69.2%, T2: 73.3%, and T3: 43.2%). At all timepoints, the majority reported their practice setting as medical school or hospital-based with no significant change over time (Wald *χ*^2^(2) = 4.54, *p* = 0.1; T1: 67.6%, T2: 73.3%, and T3: 77.7%). The mean age of participants at participation increased slightly in the T3 survey (T1: 51.9 years; T2: 51.1; T3: 56.3). A significant interaction between timepoint and specialty was found in participants’ age (Wald χ^2^(2) = 71.10, *p* < 0.001): urologists (T1:51.5 years; T2:54.3; T3:53.9) were older in T2, but younger in T3, compared to endocrinologists (T1: 52.1 years; T2: 50.0; T3:59.3). Participants’ clinical experience, as measured by the number of patients treated during their career, was slightly less during the T2 survey (T1: 50.0; T2: 42.5; T3: 50; Wald χ^2^(2) = 9.47, *p* = 0.009).

**Table 2:** Clinician survey content overview

Section	Survey contents: major components	
Introduction	Overview of survey and eligibility screener^a^	
Demographics	Clinical practice and demographic characteristics	
Clinical case scenarios	Cases	Micropenis (Primary testicular failure) Partial insensitivity syndrome (PAIS) Penile ablation
	Decisions	Gender of rearing *In your professional judgment, which sex assignment/gender of rearing would result in the best long-term quality of life outcome [‘sex assignment’ does NOT necessarily imply genital surgery]?* Surgical decision-maker *Who should decide whether genital surgery (hypospadias repair) should be performed?* Timing of surgery (lists case-specific procedures) *In your professional judgment, when should genital surgery be performed?* Timing of disclosing surgical history to patient *Genital surgery is sometimes completed at an early age such that the boy will have no memory of the procedure. If surgery had been performed at such an age in the case of this particular patient, do you think that information regarding the details of the surgery or karyotype should be disclosed to the patient? If so, when?*

Cases	Clinical case sceanrios	
Micropenis		Undervirilized 46,XY whose penis when first examined at birth could be palpated only as a thin cord of tissue and with all the suprapubic adipose tissue pushed back, the penis measured 1 cm in length. The urinary meatus terminated at the tip of the penis. Testosterone treatment (25 mg testosterone cypionate once a month for 3 months) increased penis length to 1.8 cm (or − 2.5 standard deviations below age-adjusted norms) with substantial increase in diameter. Testes were small (< 0.5 cm) and somewhat soft in consistency. At 4 days of life, before testosterone treatment was begun, FSH and LH were significantly elevated for age and testosterone was 15 ng/dL. The diagnosis was considered to be Primary Testicular Failure
Partial insensitivity syndrome (PAIS)		Undervirilized 46,XY male with 1.2 cm phallus with chordee and perineal hypospadias. Basal hormone LH and FSH level at 2 days of life were clearly elevated for age consistent with a lack of an intact feedback system. Testosterone treatment (25 mg testosterone cypionate) resulted in some redness and swelling of the penile skin, with measurement increasing slightly to about 1.7 cm. The child had no suggestion of any other problems. A presumptive diagnosis of Partial Androgen Insensitivity Syndrome was assigned
Penile ablation	[No image]	46,XY. Mishandled circumcision within the first week of life resulting in complete penile ablation (Penile Ablation). Testes were of normal size and consistency, and fully descended. LH, FSH, and testosterone were normal for a male child at 6 days of age

^a^Survey overview and instructions were included in all years; Eligibility screener was included only at T3 (2020)

A detailed description of recruitment, participation rate, and participant personal and professional characteristics have been provided elsewhere (Appendix, 10.7302/6728).

### Measures

#### Survey Development

Details of design and development of the survey are included in Appendix. The survey consisted of five sections: (1) Demographics; (2) Case Scenarios; (3) Factors Affecting Life Satisfaction; (4) Surgical Informed Consent; and (5) Mental Health Services and the DSD Team (Table 2). Data from Sections  3, 4, and 5 are not included in this paper.

#### Demographics

This section collected participants’ sociodemographic and specialty characteristics, including: (1) number of DSD cases visited per career; (2) specialty (Endocrinology/Urology); (3) practice location (United States/Canada/Mexico/Other); (4) practice setting (solo or two-physician practice/group practice/HMO/medical school or hospital-based/other patient care employment/other non-patient care employment); (5) gender (man/woman/other); and (6) participant birth year.

#### Case Scenarios

This section included descriptions of three clinical scenarios of which two were 46,XY DSD (micropenis and partial androgen insensitivity syndrome [PAIS]) and the third, a case of iatrogenic penile ablation in infancy (Table 2).^4^ After each case, participants were asked what gender of rearing they would recommend (boy/girl/other), who would they recommend to be involved in the decision-making about urogenital surgery (parents, in conjunction with physician/patient, likely during adolescence), when would they recommend performing each surgery (before 6 months/before 1 year/before school entry/during pre-adolescence [ages 6–10 years]/adolescence [11 years or older]/I would recommend against surgery); and when they would recommend disclosing details of the medical and early surgical history to the patient (disclosure before school entry [5 years]/disclosure during middle childhood [6–10 years]/disclosure during adolescence [11–17 years]/disclosure during adulthood [18 years or older]/I would recommend against disclosure). The case scenario section of the survey was designed with conditional branching and skip patterns (see Fig. 2 in Appendix, 10.7302/6728).^5^ Accordingly, responses to stem questions determined follow-up questions relevant to that choice alone: for example, those who recommended the “boy” option for gender of rearing, would only receive questions for the clinical management of a boy; questions regarding surgical timing are only asked within the subgroup of participants who recommended a particular gender of rearing (e.g., those who recommended “boy” could respond to questions regarding phalloplasty, but not vaginoplasty) and who recommended that the “parent” leads decision-making.^6^

### Procedure

Invitation letters that included an explanation of the study and survey login instructions were emailed to PES and SPU members in 2003–04 (T1), 2010–11 (T2), and 2020 (T3). Participants were promised confidentially of their responses.

### Data Analysis Plan

Participant demographic characteristics and responses to case scenarios were summarized using descriptive statistics. Trends in recommendations and their possible associations with participant characteristics (gender, age, specialty, clinical experience [as measured by the number of cases managed per career], and medical setting), were examined by Generalized Estimating Equations (GEE). Accounting for the correlation between multiple responses from the same respondent (clustering by respondent), GEE has been recommended as a method for modeling longitudinal and categorical data (Agresti, 2002). In the case of the gender of rearing question, the “other” option was not available at timepoints T1 or T2: to accommodate this change, gender of rearing was dichotomized as “boy” versus “not boy.” Continuous data (e.g., physician age, and number of cases managed over the career) were dichotomized using a median split to address outliers and categorized as “older versus younger” (cut point: birth year 1952) and “more versus less experienced” (cut point = 50 cases).

Although there were six options for timing of surgery and disclosure, the responses for timing of surgery were classified for analysis into three categories: “early” (within the first year); “late” (after first year); and a recommendation against surgery. Timing of disclosure options were similarly classified into three categories: “early” (before 18 years), “late” (after 18 years), and a recommendation against disclosure. However, the actual selections made by the respondents are preserved in Fig. 3 (timing of surgery) and Fig. 4 (timing of disclosure). Practice setting was dichotomized into “medical school or hospital-based” versus “other.” For each recommendation, the first model includes the predictor variables of survey timepoints, specialty, age, gender, practice setting, and clinical experience, and the second model includes the previous predictors in addition to the interaction between survey timepoints and specialty. Finally, the same analysis model was used in predicting gender of rearing recommendations in a subsample of participants who had participated in all three surveys. All analyses were conducted using the Statistical Package for the Social Sciences (SPSS) for Windows software, Version 28.0.

## Results

### Gender of Rearing Recommendations: Boy versus Girl versus Other (Fig. 1 and Supplementary Table 1)

#### Micropenis

Across study timepoints, most recommended rearing the newborn as a boy (T1: 94.6% [406 out of 429]; T2: 94.9% [413 out of 435]; T3: 89% [235 out of 264]), however, a slight, but significant shift to a gender other than boy was evident across timepoints (Wald χ^2^(2) = 22.43, *p* < 0.001). Very few at each timepoint recommended rearing the newborn as a girl (T1: 5.4% [23 out of 429]; T2: 5.1% [22 out of 435]; T3: 2.3% [6 out of 264]). No other predictor was statistically significant. At T3, 7.9% (9 out of 114) of endocrinologists and 9.3% (14 out of 150) of urologists chose a gender other than boy or girl.

**Fig. 1:**
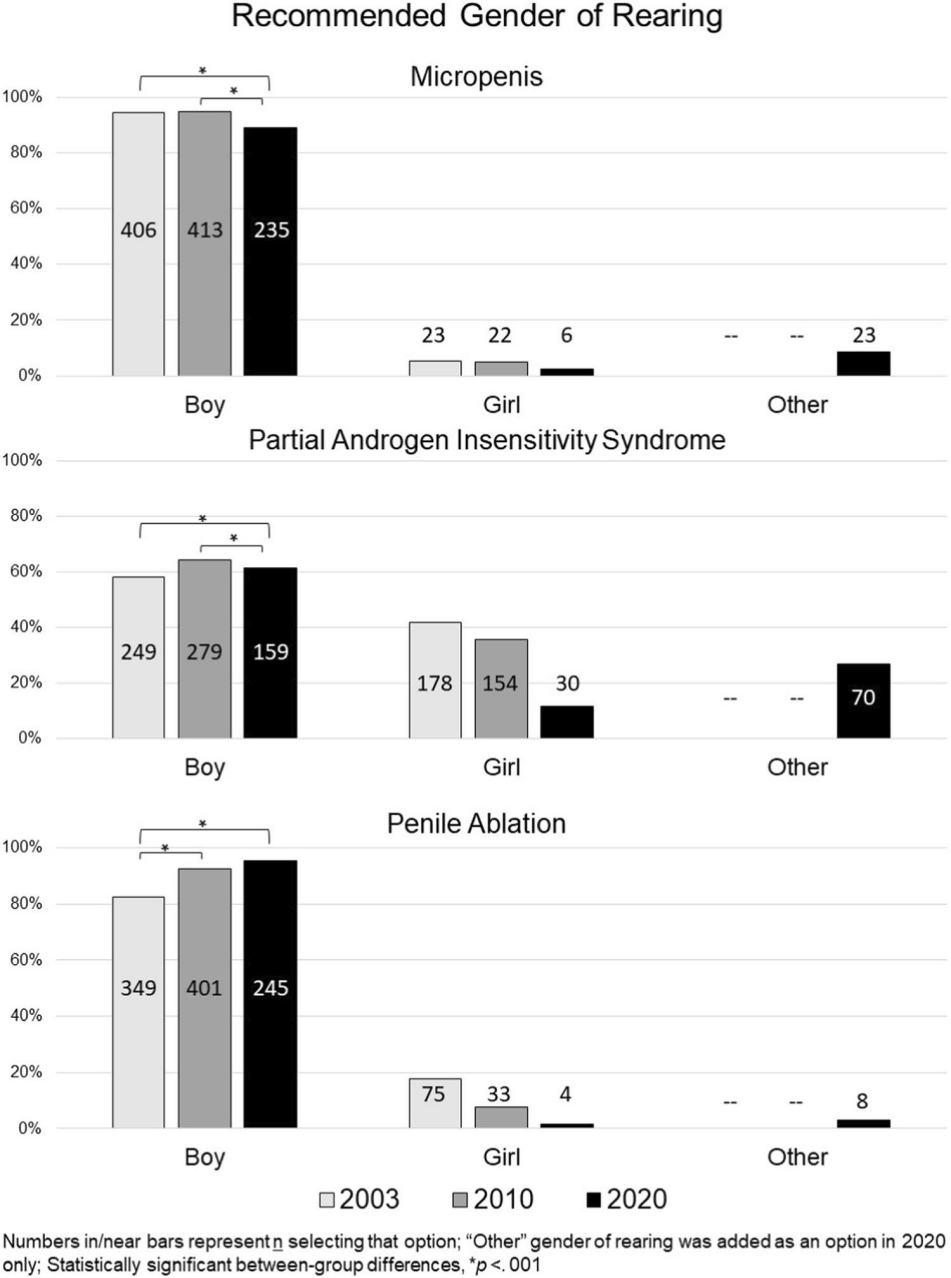
Recommendations for gender of rearing

#### Partial Androgen Insensitivity Syndrome

Across study timepoints, the majority recommended rearing the newborn as a boy (T1: 58.3% [249 out of 427]; T2: 64.4% [279 out of 433]; T3: 61.4% [159 out of 259]), and the proportion of those recommending rearing as a girl declined over time with a marked drop at the T3 (T1: 41.7% [178 out of 427]; T2: 35.6% [154 out of 433]; T3: 11.6% [30 out of 259]). Moreover, there was an interaction between specialty and survey timepoint (Wald’s χ^2^(2) = 6.81, *p* = 0.033): Across all timepoints, urologists (T1: 82.3%; T2: 85.2%; T3: 69.2%) were more likely than endocrinologists (T1: 47.8%; T2: 56.9%; T3: 51.3%) to recommend rearing the newborn as a boy. Notably, at T3, 28% (32 out of 113) of endocrinologists and 26% (38 out of 146) of urologists recommended a gender or rearing other than boy or girl.

#### Penile Ablation

Across study timepoints, most recommended rearing the newborn as a boy, and this recommendation significantly increased over time (T1: 82.3% [349 out of 424]; T2: 92.4% [401 out of 434]; T3: 94.2% [245 out of 260]; Wald’s χ^2^(2) = 13.34, *p* = 0.001). A declining group of participants recommended rearing the newborn as a girl (T1: 17.7% [75 out of 424]; T2: 7.6% [33 out of 434]; T3: 1.5% [4 out of 260]). A significant main effect was also observed for participant specialty: Urologists (T1: 90.6% [116 out of 128]; T2: 94.8% [109 out of 115]; T3: 95.9% [141 out of 147]) were more likely than endocrinologists (T1: 78.8% [233 out of 296]; T2: 91.5% [292 out of 310]; T3: 92% [104 out of 113] to recommend rearing the newborn as a boy; Wald’s χ^2^(1) = 13.451, *p* < 0.001). At T3, 4.4% (n = 5) of endocrinologists and 4.1% (n = 6) of urologists chose a gender other than boy or girl. No urologists recommended rearing the newborn as a girl at T3. Across the three timepoints, the odds of female clinicians were 2.7 times higher than male clinicians to recommend rearing the newborn as a boy (Supplementary Table 1, 10.7302/6728).

### Surgical Decision-Making: Parents versus Patients (Fig. 2 and Supplementary Table 2)

#### Micropenis

##### Recommending Rearing as Boy

Through three timepoints, a significant increase toward including the patient in surgical decision-making was detected (T1: 50.2% [203 out of 404]; T2: 58.1% [240 out of 413], and T3: 68.4% [160 out of 234], Wald’s χ^2^(2) = 18.29, *p* < 0.001). No significant difference between specialties was found.

**Fig. 2:**
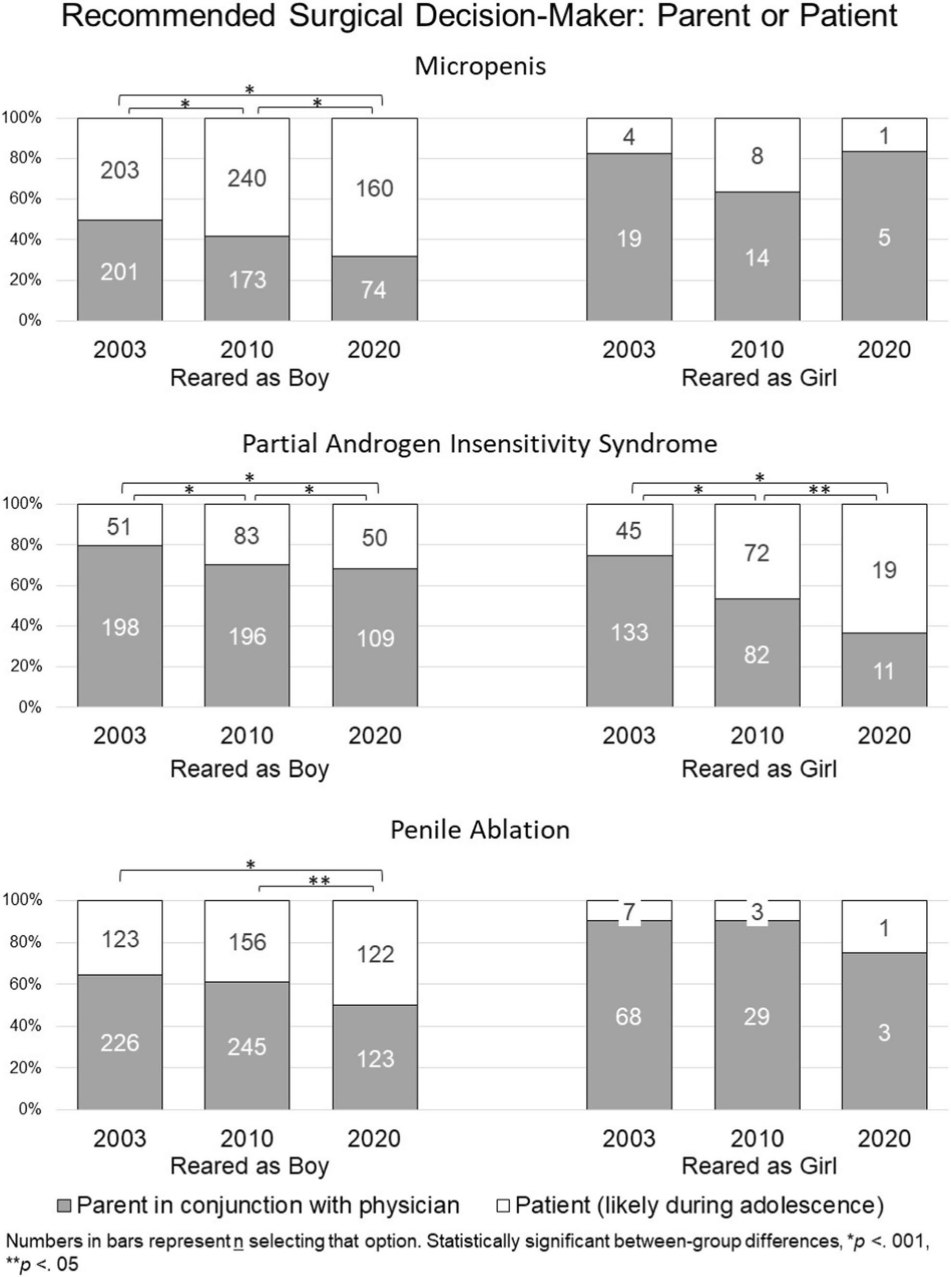
Recommendations regarding surgical decision-maker

##### Recommending Rearing as Girl

Across study timepoints, most recommended the parent (in consultation with the physician) should serve as decision-maker (T1: 82.6% [19 out of 23]; 63.6% [14 out of 22]; 83.3% [5 out of 6]) as a source of decision-making. There was no significant change over time and no significant difference between specialties.

#### Partial Androgen Insensitivity Syndrome

##### Recommending Rearing as Boy

Across study timepoints, most recommended the parents should make surgical decisions (T1: 79.5% [198 out of 249]; T2: 70.3% [196 out of 279]; T3: 68.6% [109 out of 159]); however, a statistically significant increase was observed in the proportions recommending the patient, when older, serve as decision-maker (Wald’s χ^2^(2) = 15.13, *p* < 0.001). Urologists (T1: 92.5% [99 out of 107]; T2: 89.8% [88 out of 98]; T3: 79.2% [80 out of 101]) were more likely at each timepoint to recommend that parents make the decision regarding surgery compared to endocrinologists (T1: 69.7% [99 out of 142]; T2: 59.7% [108 out of 181]; T3: 50% [29 out of 58]; Wald’s χ^2^(1) = 34.18, *p* < 0.001).

##### Recommending Rearing as Girl

A statistically significant shift toward deferring to the patient as decision-maker occurred over time (T1: 25.3% [45 out of 178]; T2: 46.8% [72 out of 154]; T3: 63.3% [19 out of 30]; Wald’s χ^2^(2) = 17.33, *p* < 0.001). No other statistically significant effects were detected.

#### Penile Ablation

##### Recommending Rearing as Boy

Across study timepoints, most participants recommended that parents make surgical decisions, but a significant increase was observed in those recommending that the decision be deferred to the patient when older (T1: 35.2% [123 out of 349]; T2: 38.9% [156 out of 401]; T3: 49.8% [122 out of 245]; Wald’s χ^2^(2) = 10.73, *p* = 0.005). A significant main effect for specialty was also found with endocrinologists (T1: 67.8% [158 out of 233]; T2: 63% [184 out of 292]; T3: 51.9% [54 out of 104]) more likely to choose parents as the source of decision-making compared to urologists (T1: 58.6% [68 out of 116]; T2: 56% [61 out of 109]; T3: 48.9% [69 out of 141]; Wald’s χ^2^(2) = 4.71, *p* = 0.03). No other statistically significant effects were detected.

##### Recommending Rearing as Girl

Across study timepoints, most recommended having the parents decide about surgery (T1: 90.7% [68 out of 75]; T2: 90.6% [29 out of 32]; T3: 75% [3 out of 4]). Significant effects were not detected for any of the predictors (Supplementary tables, 10.7302/6728).

### Surgical Timing Recommendations: Before or After One Year of Age (Fig. 3)

#### Micropenis

##### Recommending Rearing as Boy

Among those recommending parents should lead decision-making, phalloplasty after 1 year was more frequently recommended option at each timepoint (T1: 42.2% [84 out of 199]; T2: 43.4% [75 out of 173]; T3: 45.8% [33 out of 72]) than either no or late phalloplasty. No other statistically significant effects were detected.

**Fig. 3:**
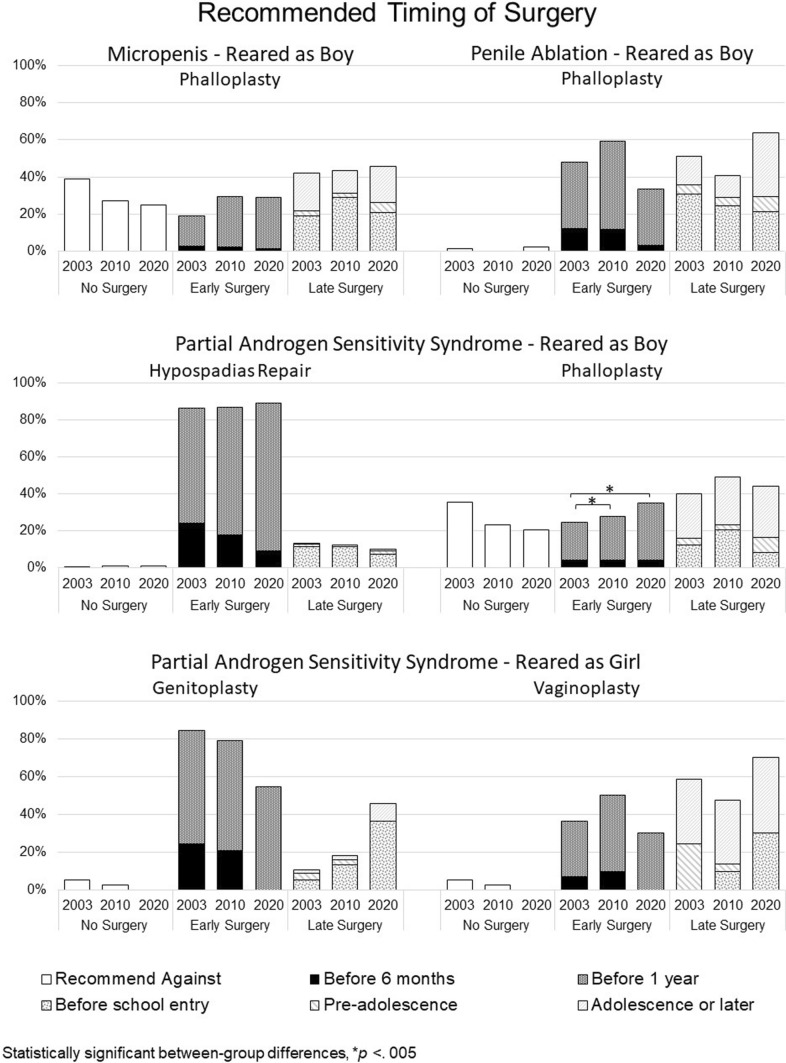
Recommendations regarding timing of surgery

##### Recommending Rearing as Girl

Across study timepoints, among those recommending parents should lead decision-making, most recommended genitoplasty be performed within the first year of life (T1: 100% [19 out of 19]; T2: 78.6% [11 out of 14]; T3: 80% [4 out of 5]). However, considering the limited sample size for those recommending this option, statistical analysis was not possible due to quasi-complete separation (Sauter & Held, 2016). A plurality of this subset of participants recommended late vaginoplasty at T1 and T2, whereas at T3 more participants encouraged early surgery (T1: 42.1% [8 out of 19]; T2: 28.6% [4 out of 14]; T3: 60% [3 out of 5]). Urologists (T1: 80% [4 out of 5]; T2: 100% [2 out of 2]; T3: 66.7% [2 out of 3]) were more likely than endocrinologists (T1: 50% [7 out of 14]; T2: 58.3% [7 out of 12]; T3: 0% [0 out of 2]; Wald’s χ^2^(1) = 6.06, *p* = 0.014) to recommend late vaginoplasty (data not shown in Fig. 3).

#### Partial Androgen Insensitivity Syndrome

##### Recommending Rearing as Boy

Across study timepoints, among those recommending parents lead decision-making, most recommended hypospadias repair be performed within the first year of life (T1: 86.3% [170 out of 197]; T2: 86.7% [170 out of 196]; T3: 89% [97 out of 109]) with no statistically significant change detected over time or between specialties. Among those in favor of parents leading decision-making, more recommended phalloplasty be performed late, at all timepoints, (T1: 40% [78 out of 195]; T2: 49% [96 out of 196]; T3: 44.3% [43 out of 97]) than either no or early phalloplasty. However, a significantly increasing proportion of these participants recommended phalloplasty be performed in the first year of life (T1: 25%; T2: 28%; T3: 35%) along with a declining trend in recommending against phalloplasty (T1: 35.4%; T2: 23.5%; T3: 20.6%; Wald’s χ^2^ (2) = 12.166, *p* = 0.002). Urologists (T1: 49% [48 out of 98]; T2: 38.6% [34 out of 88]; T3: 24.3% [17 out of 70]) were more likely than endocrinologists (T1: 21.6% [21 out of 97]; T2: 11.1% [12 out of 108]; T3: 11.1% [3 out of 27] to be opposed to phalloplasty at each timepoint (Wald’s χ^2^(1) = 14.81, *p* < 0.001). No other statistically significant effects were detected.

##### Recommending Rearing as Girl

Across study timepoints, among those recommending parents lead decision-making, most recommended early genitoplasty across all time points (T1: 84.4% [52 out of 58]; T2: 79.3% [76 out of 82]; T3: 54.5% [10 out of 11]). Neither survey timepoint nor any other factor was associated with significant differences in surgical timing recommendations. In the case of vaginoplasty, the recommendation for early surgery varied in time (T1: 36.2% [21 out of 58]; T2: 50% [41 out of 82]; T3: 30% [3 out of 10]), although not statistically significantly.

#### Penile Ablation

##### Recommending Rearing as Boy

Among those recommended parents lead decision-making, recommendations for the timing of phalloplasty varied across survey timepoints (Early surgery, T1: 47.8% [108 out of 226]; T2: 59.3% [146 out of 246]; T3: 33.6% [41 out of 122]), yet a significant trend was not discernable. Across survey timepoints, endocrinologists (T1: 55.1% [87 out of 158]; T2: 70.8% [131 out of 185]; T3: 56.6% [30 out of 53]) were more likely to recommend early surgery compared to urologists (T1: 30.9% [21 out of 68]; T2: 24.6% [15 out of 61]; T3: 15.9% [11 out of 69]; Wald’s χ^2^(1) = 45.353, *p* < 0.001). No other statistically significant effects were detected.

##### Recommending Rearing as Girl

The number of those responding to this question decreased substantially across timepoints, ruling out statistical analysis due to quasi-complete separation (Sauter & Held, 2016). Across study timepoints, among those who recommended parents lead decision-making, most recommended early genitoplasty (T1: 95.6% [65 out of 68]; T2: 86.2% [25 out of 29]; T3: 100% [3 out of 3]), and late vaginoplasty (T1: 57.4% [39 out of 68]; T2: 69% [20 out of 29]; T3: 66.7% [2 out of 3]) (data not shown in Fig. 3).

### Disclosing Early Surgery and Discordant Karyotype (Fig. 4)

#### Micropenis

##### Recommending Rearing as Boy

Across study timepoints, most recommended an early (before 18 years) disclosure of masculinizing surgery (T1: 91.1% [364 out of 400]; T2: 94.1% [387 out of 411]; T3: 96.9% [223 out of 230]) with a significant increase in percentage of those recommending so over time (Wald’s χ^2^ (2) = 10.686, *p* = 0.005). No other statistically significant effects were detected.

**Fig. 4:**
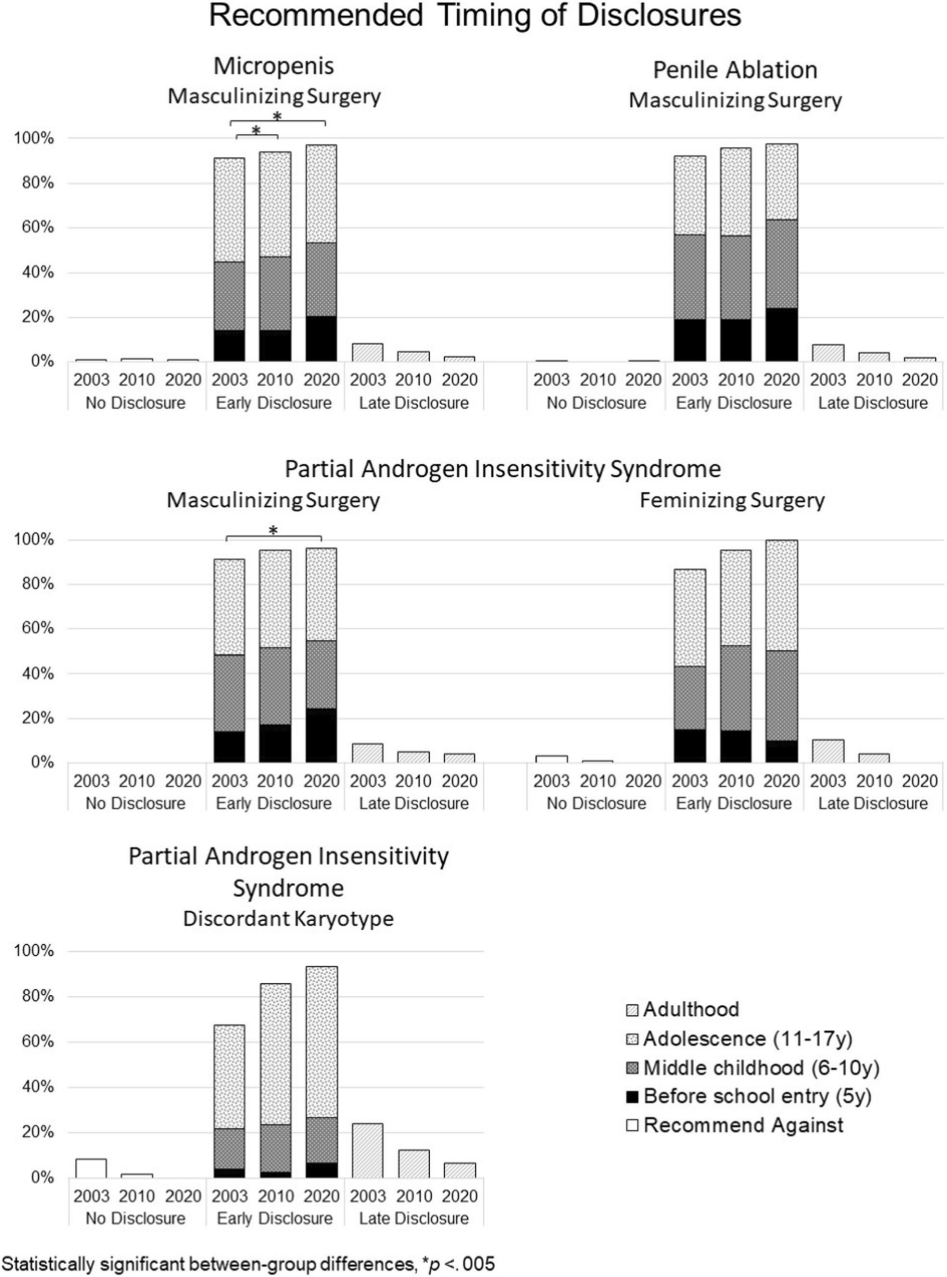
Recommendations for timing of disclosure of medical and surgical history

##### Recommending Rearing as Girl

Across study timepoints, the majority recommended early disclosure of surgery was performed (T1: 47.8% [11 out of 23]; T2: 90.9% [20 out of 22]; T3: 100% [6 out of 6]), but in case of disclosing karyotype, the recommendations varied substantially across study timepoints (Early disclosure, T1: 31.8% [7 out 22]; T2: 81.8% [18 out of 22]; T3: 66.7% [4 out of 6]). Statistically significant effects were not detected for any of the predictors (data not shown in Fig. 4).

#### Partial Androgen Insensitivity Syndrome

##### Recommending Rearing as Boy

The vast majority of participants at all 3 timepoints recommended early disclosure of penile reconstruction (T1: 91.5% [226 out of 247]; T2: 95.3% [266 out of 279]; T3: 96.2% [151 out of 157]). Significant effects were not detected for any of the predictor variables.

##### Recommending Rearing as Girl

Across study timepoints, most recommended an early disclosure that feminizing surgery (i.e., genitoplasty) had occurred (T1: 86.9% [153 out of 176]; T2: 95.5% [147 out of 154]; T3: 100% [30 out of 30]) and that the patient’s karyotype was 46,XY (T1: 67.4% [118 out of 175]; T2: 85.7% [132 out of 154]; T3: 93.3% [28 out of 30]). Again, none of the predictor variables were found to be statistically significant.

#### Penile Ablation

##### Recommending Rearing as Boy

Across study timepoints, virtually all participants recommended early disclosure of penile reconstruction with a statistically significant increase observed across timepoints (T1: 91.9% [310 out of 347]; T2: 95.8% [384 out of 410]; T3: 97.6% [239 out of 245]; Wald’s χ^2^(2) = 6.987, *p* = 0.03). No other statistically significant effects were detected (data not shown in Fig. 4).

##### Recommending Rearing as Girl

Similar to those recommending a male gender of rearing, most recommended early disclosure to the patient reared as a girl that surgery had occurred at a young age (T1: 86.7% [65 out of 75]; T2: 81.3% [26 out of 32]; T3: 100% [4 out of 4]). Lower proportions recommended early sharing of 46,XY karyotype (T1: 57.3% [43 out of 75]; T2: 59.4% [19 out of 32]; T3: 75% [3 out of 4]). Statistically significant effects of survey timepoint were not detected for disclosure of either element. At each timepoint endocrinologists were more likely to recommend early disclosure of surgery (T1: 87.3% [55 out of 63]; T2: 88.5% [23 out of 26]; T3: 100% [4 out of 4]) and karyotype (T1: 55.6%; T2: 65.4%; T3: 75%) compared to urologists (surgery, T1: 83.3% [65 out of 75]; T2: 50% [3 out of 6]; T3: 0% [0 out of 0]; Wald’s χ^2^(1) = 10.048, *p* = 0.002) and (karyotype, T1:66.7%; T2: 33.3%; T3: 0%; Wald’s χ^2^(1) = 4.61, *p* = 0.032) (data not shown in Fig. 4).

### Subsample Participating in All Three Surveys

A total of 82 participants participated in all three surveys. The median birth year was 1957, 57.3% were members of PES, and 74.2% identified as men. In the case of micropenis, although 71 participants (out of 81, 88%) invariably recommended “boy” as the gender of rearing at each timepoint, the proportion of the total subsample making this recommendation declined slightly over time (T1: 97.6%; T2: 95.1%; T3: 88.9%; Wald’s χ^2^(2) = 6.27, *p* = 0.043). From 2003 to 2010, 2% (2 out of 81; *n* = 1 with missing data) changed their gender of rearing recommendation (both from “boy” to “girl”), and from 2010 to 2020, 10% (8 out of 81) changed their gender recommendation, mostly from “boy” to “other” (n = 4).

In the case of PAIS, 35 (out of 81, 43%) and 5 (out of 81, 6%) participants consistently recommended “boy” and “girl” as the gender of rearing, respectively. Although at T1, 36% had recommended “girl,” a significant decline in this recommendation was observed across timepoints (T1: 36%; T2: 24.7%; T3: 16%; Wald’s χ^2^(2) = 12.762, *p* = 0.002). From 2003 to 2010, 24% (21 out of 81) changed their recommendations, predominantly from “girl” to “boy” (n = 15), and from 2010 to 2020, 37% (30 out of 81) changed their recommendations from “boy” to “other” (n = 11) and “girl” to “boy” (n = 8).

In the case of penile ablation, 64 (out of 79, 81%) consistently recommended “boy” as the gender of rearing at each study timepoint, (“boy” as gender of rearing, T1: 86.3%; T2: 93.8%; T3: 93.8%; ns). Only one participant recommended a female gender of rearing at all timepoints. From 2003 to 2010, 10% (8 out of 79) changed their recommendation, mostly from “girl” to “boy” (n = 7), and from 2010 to 2020, 10% (6 out of 79) changed their recommendations, again mostly from “girl” to “boy” (n = 4) and “boy” to “other” (n = 2).

## Discussion

At three survey timepoints, spanning two decades, this survey assessed the clinical recommendations of pediatric endocrinologists and urologists for individuals 46,XY DSD using three clinical scenarios (micropenis, PAIS and iatrogenic penile ablation). The findings in all areas of management were mostly in line with our hypotheses, and some in apparent disagreement with the optimal gender policy. In all cases, a minority recommended rearing the newborn as a girl and the proportion doing so declined across survey timepoints. When the option of recommending the gender “other” (intersex or non-binary) was added in T3, the proportion recommending this option was comparable to that recommending rearing as a girl. The next decision point after gender of rearing involved consideration of genital surgery: A significant trend was observed for some cases to defer decision making to the patient when they were older. Within the subset of participants who recommended that parents lead surgical decision making, recommendations regarding timing of surgery varied based on the gender of patient and type of surgery: the only significant change was an increasing proportion, from T1 to T3, in recommending early phalloplasty for newborns with PAIS who were reared as a boy. Compared to endocrinologists, urologists were significantly less likely to recommend early phalloplasty in boys with PAIS and boys with penile ablation. Independent of the condition and gender assignment, most participants recommended an early disclosure of both medical and surgical details to the patient, and this recommendation became more likely with successive waves of the survey.

### Gender Assignment

According to the optimal gender policy (Money et al., 1955a, 1955b), all three 46,XY cases presented would presumably have been assigned as girls and their genitalia would have been surgically reconstructed as early as possible to eliminate doubts, initially in the parents and later the patient (Meyer-Bahlburg, 2005). Very few participants in our study followed this approach, and the majority recommended “boy” as the gender of rearing. Nevertheless, long-term follow-up studies suggest that neither recommendation (girl nor boy) is guaranteed to yield a stable gender identity throughout the lifespan. A recent review of patients with PAIS reported that 12% (14 out of 113 cases) of those reared as girls and 25% (7 out of 28 cases) of those reared as boys, developed gender dysphoria later in life (Babu & Shah, 2021). Similarly, out of 7 cases reported of penile ablation in the newborn period and physician-recommended reassignment as female, three were either subsequently dysphoric or self-initiated gender change to male and four were living as women (Meyer-Bahlburg, 2005). One of those assigned female at birth who later identified as man, was the John/Joan case (Colapinto, 1997; Money, 1975).

In all three clinical scenarios, consistent with our hypothesis regarding a deviation from the optimal gender policy, the proportion of individuals suggesting raising the child as a “girl” decreased. Specifically, in the instance of penile ablation, there was an increase in the percentage of individuals recommending “boy” as the gender of rearing. These trends may reflect a growing tendency among survey participants to believe in the importance of prenatal androgenization effects on the fetal brain and gender development (Diamond, 2009; Hines, 2020; Imperato-McGinley et al., 1974; Reiner & Gearhart, 2004). The findings regarding gender of rearing recommendations by North American DSD specialists mirrors a report stemming from the International Disorder of Sex Development (I-DSD) Registry. That study suggested a secular trend in favor of rearing as boys individuals born with a variety of 46,XY DSD (Kolesinska et al., 2014). In both this North American and the largely European I-DSD registry study, the shift away from the recommendations of the optimal gender policy already preceded the publication of the 2006 Consensus Statement (Lee et al., 2006).

Urologists in our surveys were more likely than endocrinologists to recommend rearing newborns with PAIS and penile ablation as boys. The tendency of urologists for recommending a male gender has also been reported in a survey on gender assignment for newborns with 46,XY cloacal exstrophy and a 6-year follow-up study. In both surveys, the most important factor given by urologist participants for recommending a male gender assignment was “the likelihood of brain imprinting by androgens” (Diamond et al., 2006, 2011).

Another important observation from the T3 timepoint (2020) was that a meaningful minority of clinicians recommended rearing the newborn with a gender other than boy or girl (micropenis: n = 23 [8.7%]; PAIS: n = 70 [27%]; penile ablation: n = 11 [4.2%]). Although the survey did not inquire about the rationale for the gender of rearing recommendation, it is plausible that these clinicians anticipated that the affected individual, whether reared as either boy or girl, would ultimately experience gender dysphoria. Yet, we could not find any long-term study on the psychosexual outcome of children reared from birth as non-binary or with an intersex identity. Based on at least one rather old survey of a clinical sample of adults with 46,XY regarding attitudes to clinical management policies, 85% did not agree with a third gender policy which had been defined as “…culture should permit a third gender in addition to male and female so that children born with unfinished sex organs (ambiguous genitalia) would not have to be declared male or female and would not have to have operations performed on their sex organs” (Meyer-Bahlburg et al., 2004, p. 1617).

### Surgical Decision-Maker and Surgical Timing

In 2003, the majority of clinicians recommended that parents serve as the decision-makers for surgical interventions, but this trended significantly in successive survey waves toward designating the patient as decision-maker. It is important to note that including patients in decision making necessarily entails postponing surgical interventions. This trend toward including patients may reflect the impact of reports in the medical literature (Creighton et al., 2001; Crouch et al., 2008) and mainstream media (Compton, 2021; Davis, 2022) of unsatisfactory outcomes in patients with DSD who had received surgical interventions as infants. It could also be due to legal and ethical arguments raised on the basis of patient bodily autonomy (Feder & Dreger, 2016; Greenberg, 2017; Human Rights Watch, 2017; Kon, 2015). Independent of why an increasing number of clinicians are recommending a shift in decision making from parents to patients, this trend is not in line with the opinions of the majority of adults with a DSD who as infants or young children received urogenital surgery: based on a recent review of surveys of patients’ preferences for timing of genital surgery, a clear majority preferred an early intervention before age of consent (Meyer-Bahlburg, 2022).

A common subclassification of 46,XY DSD is based on whether prenatal androgen effects are present or not. A recent multicenter European study found there are differences between these subgroups: 66% of those with genital signs of androgen effects (e.g., PAIS, 17βHSD3, 5αRD2) preferred early interventions (infancy or childhood), but only 15% of those without androgens effects (e.g., complete androgen insensitivity syndrome, complete gonadal dysgenesis) indicated so (Bennecke et al., 2021). Moreover, among those with 46,XY DSD and signs of prenatal androgen effects, those reared as boys were more likely to prefer an early genital surgery (75%: 44% before 3 years of age; 31% additional before 12 years of age) compared to those reared as girls (45%: 26% before 3 years of age; plus 19% before 12 years of age) (Bennecke et al., 2021). Preferences of these patients are in contrast with the increasing tendency observed among the participants in our surveys to recommend surgical delay.

Urologists were less likely than endocrinologists to recommend early phalloplasty for cases of PAIS and penile ablation. The tendency among urologists to delay such surgeries may have originated with their recognition of the challenges of creating a neophallus and the inadvisability of planning for serial procedures so that the size of the phallus matches the individual’s chronological age. In contrast, focus on the possible harm to psychosocial and psychosexual development of being reared as a boy and having a very atypical phallus, or none at all, may have led endocrinologists to opt for performing phalloplasty at younger ages (Gardner & Sandberg, 2018).

### Disclosure

The majority of survey respondents, at all timepoints, for all cases, and regardless of suggested gender of rearing, recommended early disclosure of medical findings and surgical history to the patient. While a substantial minority of clinicians at T1 (2003) had recommended against disclosure (especially in situations where the gender of rearing was discordant with karyotype), only three participants at T3 (2020) recommended against disclosure across all case scenarios. The recommendations of our survey participants are consistent with the principal of patient-centered care that emphasizes openness regarding details of diagnosis and treatment (Institute of Medicine Committee on Quality of Health Care in America, 2001). Historically, the practice of withholding information from children about their medical conditions–whether in the management of DSD or other chronic pediatric medical conditions–has been based on the belief that they would be ill-equipped to understand and emotionally cope with such information (American Academy of Pediatrics, 1999; Sisk et al., 2016). The hesitancy to be open with the child about their condition can also originate with the child’s family (Rolston et al., 2015; Traino et al., 2022). Our current findings, uniformly recommending early disclosure, is also in line with findings showing that self-disclosure or condition openness to others is associated with better mental health, lower anxiety and depression (van de Grift, 2023).

### Limitations

One key limitation of this study is whether responses to vignette-based clinician surveys accurately reflect “real world” decision making. Considering the powerful impact of social activism and patient advocacy, one might speculate that responses in such surveys may be biased by perceived social desirability. The intersex advocacy movement, arising in the early 1990s, called for an altered paradigm of care that emphasized addressing caregivers’ emotional distress, developmentally-sensitive and comprehensive education of the person with the DSD, and deferral of elective surgical interventions (Intersex Society of North America, 2008). Clinical management in DSD remains in a state of flux, with disagreements within and between healthcare provider, advocacy, and patient communities regarding what constitutes optimal care. Evidence of these controversies exists in the medical literature (Diamond & Garland, 2014), social media (interACT: Advocates for Intersex Youth, n.d.; Organization Intersex International, n.d.), deliberations of human rights organizations (Human Rights Watch, 2017; Méndez & United Nations Human Rights Council, 2013), the U.S. Department of State (U.S. Department of State, October 6, 2023) and courts of law (Ghorayshi, August 5, 2015; Ghorayshi, July 26, 2017).

Because of such controversies and their potential influences on survey responses, future study designs should include assessments of real-life decisions in clinical management of DSD via retrospective and/or prospective medical chart review (Goodman et al., 2022). At least one study has provided evidence indicating a tendency to delay genitoplasty in actual clinical settings: A chart review study at a singular Midwestern tertiary care medical center observed a consistent decrease in the rate of clitoroplasty among patients with congenital adrenal hyperplasia between 1979 and 2013 (Schoer et al., 2018).

### Conclusions

Although considerable variability in recommendations was observed, certain trends were apparent.The majority of key clinician stakeholders do not follow the optimal gender policy approach to DSD care. There are growing tendencies toward postponing surgical interventions to allow for the patient to be directly involved in the decision-making process, and earlier disclosure of medical and surgical histories. Although the benefits of early disclosure have been emphasized and demonstrated in studies in DSD and other chronic pediatric conditions, it is yet unknown whether the increasing trend to have patients serve as primary decision makers, necessarily delaying surgery, will be associated with greater aggregate benefit or harm.

### Supplementary Information

Below is the link to the electronic supplementary material.

Supplementary file1 (PDF 256 KB)

Supplementary file2 (PDF 436 KB)

## Data Availability

Data are available upon request.

## Footnotes

1 Historically, the term used was “sex assignment” (Money et al., 1957).

2 The Lawson Wilkins Pediatric Endocrine Society (LWPES) was founded in 1972; in 2010, the name of the Society was changed to the Pediatric Endocrine Society (PES).

3 Among the very few members of PES and SPU practicing in Mexico, none participated at any survey timepoint.

4 The scenarios did not include a case of XY cloacal exstrophy. Although it would have been desirable to include such a vignette because of the challenge to female gender assignment, the key paper recommending male gender assignment was published only after the first wave of the current study (Reiner & Gearhart, 2004).

5 This option was available only at T3.

6 During the peer-review process, an unintentional asymmetry was discovered in the wording of the item dealing with disclosure of surgery to the child if surgery occurred at an age such that the child would likely have no memory of the procedure. The inconsistency appears in the third item following the stem question about gender of rearing: for those participants choosing a male gender of rearing, the disclosure item used “boy,” whereas the parallel item for those being reared as a girl, used “child.”

## Code Availability

Code is available upon request.
