# Supplementary material for: Recommendations for 46,XY Disorders/Differences of Sex Development Across Two Decades: Insights from North American Pediatric Endocrinologists and Urologists
Source: Arch Sex Behav. 2024 Jul 22;53(8):2939–56. doi: 10.1007/s10508-024-02942-1 (PMC11335971; doi:10.1007/s10508-024-02942-1)
Supplement: Supplementary file 1 — Supplementary file1 (PDF 256 KB) [file 10508_2024_2942_MOESM1_ESM.pdf]

Supplementary Table 1. Likelihood of recommending rearing as a boy across different timepoints and participant demographic characteristics

Comparison		Micropenis				PAIS				Penile Ablation			
		**OR***	**Lower**	**Upper**	***p***	**OR**	**Lower**	**Upper**	***p***	**OR**	**Lower**	**Upper**	***p***
**Year of survey administration**	2003 to 2020	3.377	1.934	5.898	<.001	1.436	1.007	2.048	0.045	0.474	0.253	0.885	0.019
	2010 to 2020	2.856	1.751	4.66	<.001	1.807	1.3	2.511	<.001	1.042	0.556	1.954	NS
	2010 to 2003	0.846	0.528	1.354	NS	0.795	0.613	1.030	NS	2.2	1.405	3.446	<.001
**Specialty**	PES to SPU	1.002	0.536	1.872	NS	0.283	0.201	0.4	<.001	0.354	0.203	0.617	<.001
**Gender**	Male to Female	1.444	0.794	2.625	NS	1.196	0.867	1.648	NS	0.374	0.222	0.629	<.001
**Age**[a]	Young to Old	2.346	1.298	4.241	0.005	1.555	1.111	2.178	0.010	2.322	1.446	3.729	<.001
**Experience**[b]	Less to More	0.823	0.503	1.349	NS	1.074	0.824	1.399	NS	0.947	0.618	1.453	NS
**Practice Setting**	Medical School or Hospital to Other	0.596	0.348	1.020	NS	0.825	0.61	1.114	NS	0.952	0.58	1.561	NS

Abbreviations: PAIS = partial androgen insensitivity syndrome, OR = Odds ratio; PES = Pediatric Endocrine Society; SPU = Societies for Pediatric Urology

[a] Median split was used to categorize participants into the younger and older age groups

[b] Median split of cases seen over one's career was used to categorize participants into the lesser and more experienced groups

* The likelihood of recommending rearing as a boy, in the first category (e.g., PES) compared to the second category (e.g., SPU)

Supplementary Table 2. Likelihood of recommending the patient lead surgical decision-making across different timepoints and participant demographic characteristics

		**Micropenis**				**PAIS**								**Penile Ablation**			
**Comparison**		**Reared as boy**				**Reared as girl**				**Reared as boy**				**Reared as boy**			
		**OR***	**Lower**	**Upper**	***p***	**OR**	**Lower**	**Upper**	***p***	**OR**	**Lower**	**Upper**	***p***	**OR**	**Lower**	**Upper**	***p***
**Year of survey administration**	2003 to 2020	0.466	0.328	0.661	<.001	0.209	0.093	0.467	<.001	0.365	0.22	0.607	<.001	0.561	0.396	0.796	0.001
	2010 to 2020	0.612	0.44	0.852	0.004	0.42	0.199	0.886	0.023	0.553	0.354	0.862	0.009	0.679	0.492	0.936	0.018
	2010 to 2003	1.314	1.011	1.707	0.041	2.015	1.29	3.148	0.002	1.512	1.021	2.239	0.039	1.209	0.9	1.624	NS
**Specialty**	PES to SPU	0.817	0.592	1.128	NS	1.264	0.62	2.578	NS	4.348	2.657	7.117	<.001	0.694	0.5	0.965	0.03
**Gender**	Male to Female	0.551	0.399	0.76	<.001	0.897	0.527	1.528	NS	0.776	0.5	1.204	NS	0.776	0.564	1.069	NS
**Age**[a]	Young to Old	0.988	0.709	1.377	NS	1.373	0.798	2.364	NS	0.966	0.588	1.587	NS	0.865	0.602	1.243	NS
**Experience**[b]	Less to More	1.317	1.008	1.721	0.044	1.654	1.092	2.505	0.018	1.084	0.744	1.578	NS	0.949	0.718	1.253	NS
**Practice Setting**	Medical School or Hospital to Other	1.391	1.030	1.879	0.031	.902	.539	1.507	NS	.964	.624	1.490	NS	1.229	.898	1.681	NS

Abbreviations: PAIS = partial androgen insensitivity syndrome; OR = Odds ratio; PES = Pediatric Endocrine Society; SPU = Societies for Pediatric Urology, OR = Odds ratio; NS = Not Significant; PES = Pediatric Endocrine Society; SPU = Societies for Pediatric Urology

a Median split was used to categorize participants into the younger and older age groups

b Median split of cases seen over one's career was used to categorize participants into the lesser and more experienced groups

* The likelihood of recommending the patient lead surgical decision-making in the first category (e.g., PES) compared to the second category (e.g., SPU).

Due to the small number of participants recommending female gender assignment for the micropenis and penile ablation cases, the analyses are not shown.
