# Supplementary material for: Recommendations for 46,XY Disorders/Differences of Sex Development Across Two Decades: Insights from North American Pediatric Endocrinologists and Urologists
Source: Arch Sex Behav. 2024 Jul 22;53(8):2939–56. doi: 10.1007/s10508-024-02942-1 (PMC11335971; doi:10.1007/s10508-024-02942-1)
Supplement: Supplementary file 2 — Supplementary file2 (PDF 436 KB) [file 10508_2024_2942_MOESM2_ESM.pdf]

Recommendations for 46,XY Disorders/Differences of Sex Development (DSD) Across Two Decades: Insights from North American Pediatric Endocrinologists and Urologists

Behzad Sorouri Khorashad[a*]
Melissa Gardner[a*]
Peter A. Lee[b]
Barry A. Kogan[c]
David E. Sandberg[a,d]

Affiliations

[a] Susan B. Meister Child Health Evaluation and Research (CHEAR) Center, Department of Pediatrics, University of Michigan Medical School, Ann Arbor, MI 48109 USA

[b] Department of Pediatrics, Penn State College of Medicine, Hershey, PA 17033, USA

[c] Chair, Department of Urology, Albany Medical College and Center, 23 Hackett Blvd. Albany, NY, 12208 USA

[d] Division of Pediatric Psychology, Department of Pediatrics, University of Michigan Medical School, Ann Arbor, MI 48109 USA

[*] denotes shared first authorship

Short Title: N Amer DSD Clin Survey: 46,XY Care

Corresponding Author:
David E. Sandberg, PhD
Child Health Evaluation and Research (CHEAR) Center
Michigan Medicine
300 North Ingalls, Ann Arbor, Michigan 48109-5456
Phone: 734-615-1958
Fax: 734-264-2599
E-mail: dsandber@med.umich.edu

Abbreviations. partial androgen insensitivity syndrome (PAIS), PES (Pediatric Endocrine Society), SPU (Societies for Pediatric Urology), clinical practice guidelines (CPG), disorders/differences of sex development (DSD)

Key words: partial androgen insensitivity syndrome (PAIS), micropenis, penile ablation, differences of sex development, gender assignment, clinical practice guidelines

DSD Clinician Survey: Survey and Participant Profile
46,XY Clinical Case Presentations

Summary

A survey of pediatric endocrinologists and urologists on clinical management practices regarding disorders (differences) of sex development / intersex conditions was administered at three timepoints. Participants were recruited from membership rosters of two North American-based professional societies: the (Lawson-Wilkins) Pediatric Endocrine Society and the Societies for Pediatric Urology. Members were sent invitations to complete the online survey at three timepoints: three years prior to publication of the 2006 "Consensus statement on management of intersex disorders," four years following it, and four years following publication of the 2020 Consensus statement update.

Survey Development

Initial Survey Development

Provisional survey items were generated based on a literature review and focus groups conducted by conference call. Focus groups were convened to identify themes pertinent to the investigation and canvass opinion regarding optimal survey administration format. Focus group participants included 16 junior and senior members of the (Lawson-Wilkins)[1] Pediatric Endocrine Society (PES) and the Societies for Pediatric Urology (SPU) nominated for participation by colleagues who thought their opinions would be particularly informative; a geographically diverse sample was sought. Web-based administration to facilitate recruitment was the consensus of focus group participants. A preliminary survey was pilot-tested with a subgroup of focus group members with other members checking for comprehensiveness of content coverage and survey response options.

The final version of the Time 1 (T1) Intersex Survey was administered in 2003-2004 and comprised five sections: 1) *Case Presentations,* 2) *Factors Affecting Life Satisfaction*, 3) *Surgical Informed Consent*, 4) *Mental Health Services and the DSD Team*, and 5) *Demographics*.

This document provides a detailed description of the survey and participant recruitment pertaining to the 46,XY case presentations in 2003-2004 as well as two subsequent survey administrations in 2010-2011 and 2020.

Three 46,XY cases were presented: two 46,XY DSD and one case of penile ablation occurring in the first week of life. Penile ablation is not a DSD, but was included in the survey as the condition has been used to inform theory development and clinical management of DSD.

Subsequent Survey Iterations

Items in the T1 survey were edited over time reflecting terminology changes. Use of earlier data collection and analyses guided later data collection. Limited changes were made to item presentation, wording, and order. When considering changes, goals were to limit the extent of changes made to allow for direct comparisons across time.

[1] Founded in 1972, the Lawson Wilkins Pediatric Endocrine Society (LWPES) formally changed its name to the Pediatric Endocrine Society (PES) in 2010.

Revised surveys, T2 and T3, were administered in 2010-2011 and 2020, respectively.

When data collection spanned >1 year, the first year in which data collection occurred is used in the label. These are hereafter referred to as the T1 2003, T2 2010, and T3 2020 surveys.

Edits to 46XY and Demographic items over time:

- Terminology changed from Intersex to DSD. In 2003, *intersex* applied to the medical condition; it was replaced by *disorders of sex development* following the 2006 Consensus Statement; by 2020, use of the term *intersex* re-emerged, but carried a different connotation for some - applying more to an identity than a medical condition, per se. Additionally, by 2020 the word *disorder* was viewed negatively by some who supported the term *difference of sex development*. Changes in wording were intended to maintain a focus on the same set of medical conditions, despite changes in vernacular used to describe these conditions.
    - T1 2003: "Intersex" and "Intersexuality"
    - T2 2010: "Disorder of Sex Development (DSD)"
    - T3 2020: "Disorders/differences of sex development (DSD)"
- Use of earlier data collection and analyses to guide later data collection – item order
    - T1 2003 began with case presentations and ended with demographics
    - T2 2010 began with demographics
    - T3 2020 began with introduction, then demographics; the introduction included a self-administered eligibility screen and opt-out with pre-populated reasons for opting out
    - T3 2020 included an "other" option for recommended gender of rearing (Case Presentation) and for participant gender (Demographics)

Survey Components

Table 1. 46,XY-focused survey components

Section	Contents: Major Components
**Introduction**	Overview of survey<br>Eligibility screener – 2020 only
**Demographics**	Clinical practice and demographic characteristics
**Clinical Case Presentations**	Cases:<br>1. Micropenis<br>2. Partial Androgen Insensitivity Syndrome<br>3. Penile Ablation<br>Decisions:<br>1. Gender of rearing<br>2. Who makes the decision about genital surgery<br>3. Timing of surgery (lists case-specific procedures)<br>4. Timing of disclosing early surgical procedures to patient<br>5. Timing of disclosing discordant karyotype to patient

Table 2. 46,XY-focused survey component order

Section	T1: 2003	T2: 2010	T3: 2020
1	Introduction	Introduction	Introduction
2	Clinical Case Presentations	Demographics	Demographics
3	Demographics	Clinical Case Presentations	Clinical Case Presentations

Figure 1. Branching and skip logic used in survey administration

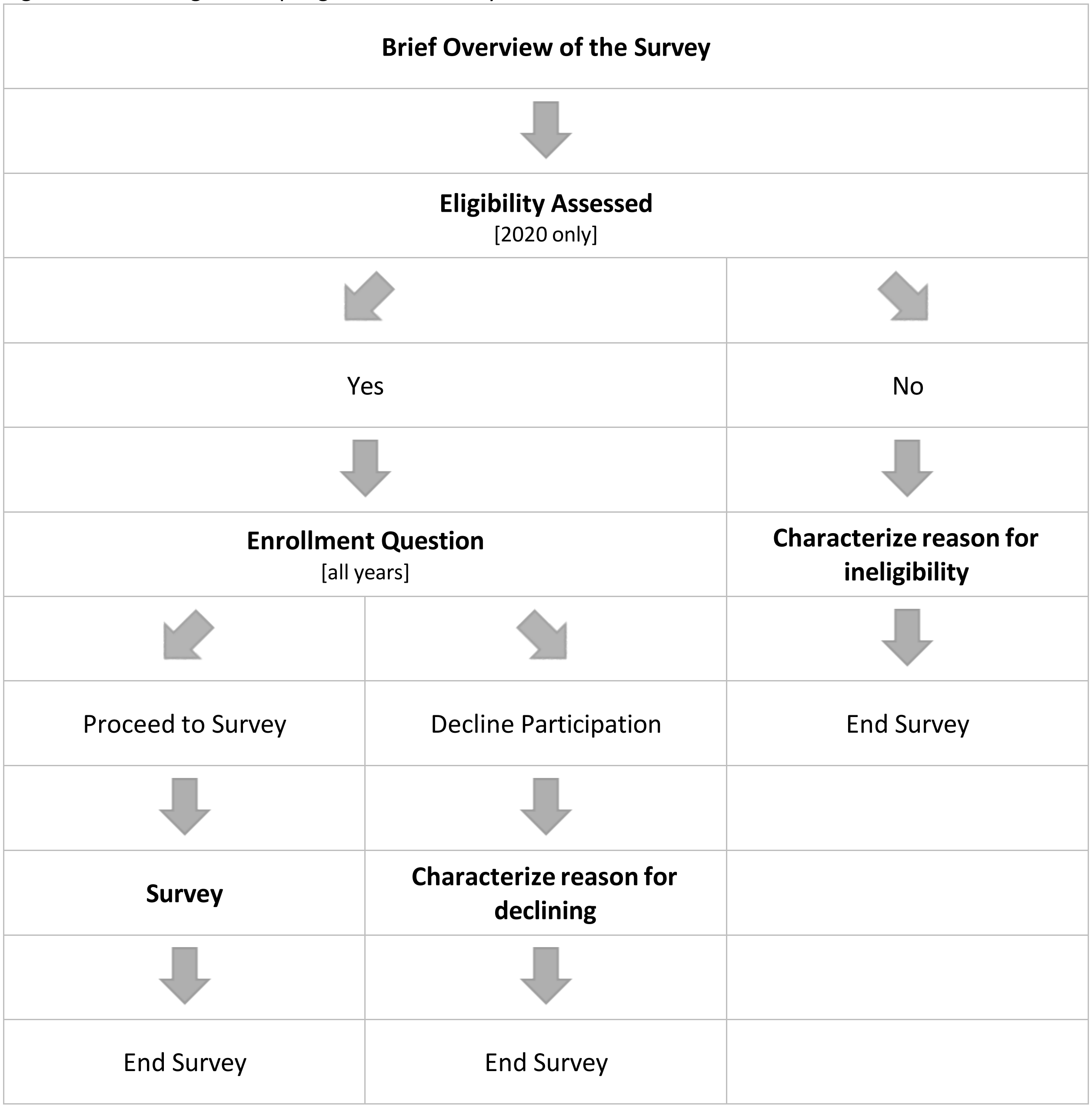

Figure 2: Branching and skip logic used in the clinical case presentation section

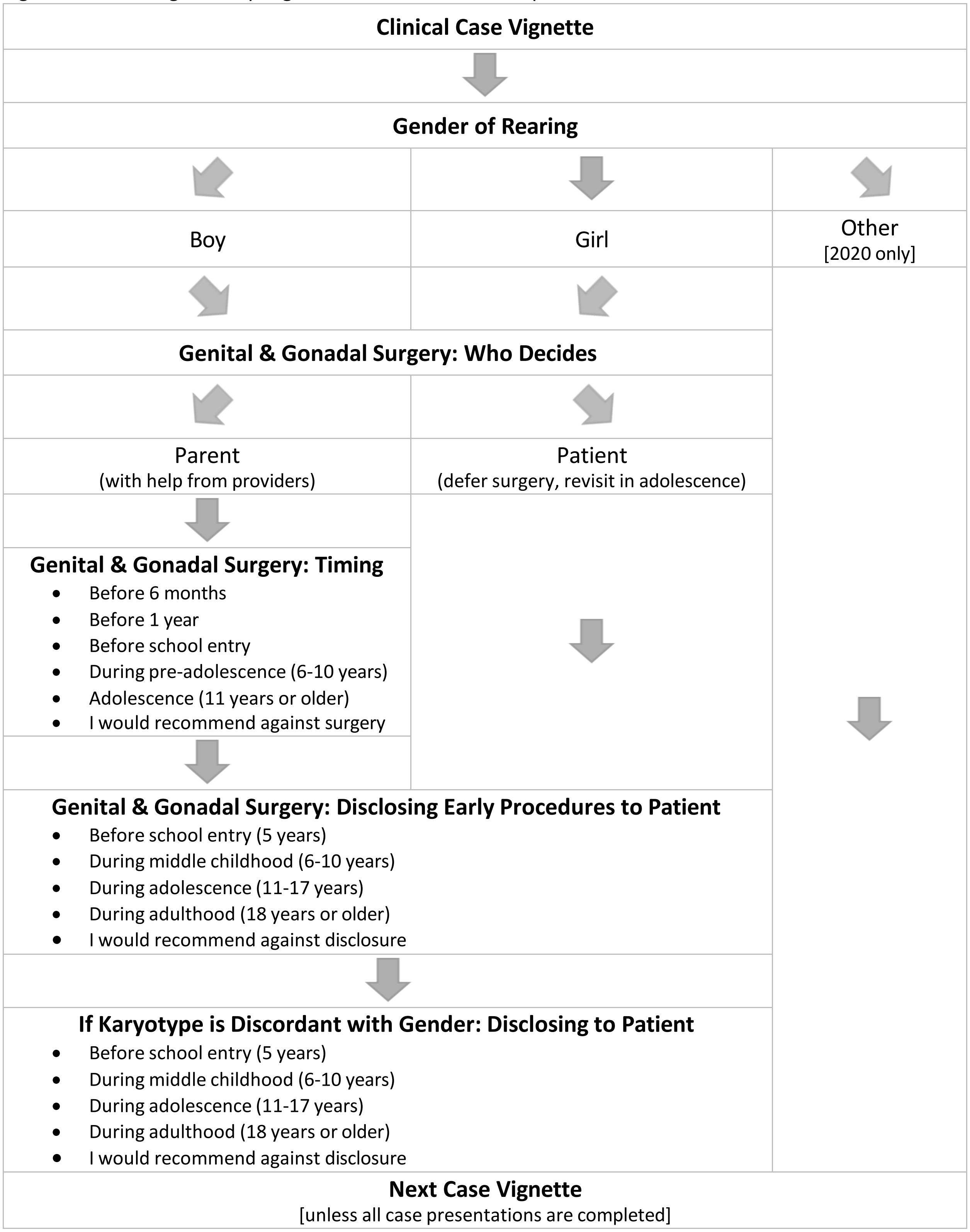

Survey Items

Notes: This survey was administered online; branching and skip logic were employed. Instances of branching and skip logic are shown below, with logic described in bracketed sections. Additional changes specific to one or more years are also noted in bracketed sections. The order of items presented below reflects that used in the 2020 survey.

Introduction: Eligibility [2020 only]

You were selected to complete this survey due to membership in the [relevant society name is displayed].

Disorders/differences of sex development (DSD) are "congenital conditions in which development of chromosomal, gonadal, or anatomical sex is atypical."

We are aware that not every society member is a practicing clinician working the area of DSD. You can indicate below whether or not you provide care to these patients or if you do not want to participate in the study. By either completing the survey or declining participation, you will not receive any more follow-up reminders to participate.

[2020 only]
Eligibility to participate:
Have you been involved in the clinical management of at least one patient with DSD?

- ☐ Yes
- ☐ No

- Yes [proceed to Enrollment question]
- [if selected "no"]
  - Please complete this last question so we can describe non-participants in any publication.
  - Are you (please select all that apply):
    - In clinical practice, with a different patient population (e.g., diabetes…)
    - In research
    - In teaching
    - In admin
    - Retired
    - Other
      - Other text
  - Thank you for your time

[all years]
Enrollment question:
- ☐ Proceed to Survey
- ☐ Decline Participation

- Yes [proceed to survey]
- [2010, 2020: if selected "decline participation"]
    - So that we can describe non-participants, are you (please select all that apply):
        - In clinical practice with DSD patients
        - In clinical practice, with a different patient population (e.g., diabetes…)
        - In research
        - In teaching
        - In admin
        - Retired
        - Other: [Write-in test response]
    - Thank you for your time

DEMOGRAPHICS

Please describe some characteristics of your practice. The data will be used ONLY for this research study. All responses are strictly confidential. Only averaged data from respondents will be utilized. No information from individual respondents will be provided to any person, group, or agency.

About how many children/adolescents/adults do you see annually who were born with DSD?
2003: About how many children/adolescents/adults do you see annually who were born with intersexuality?
________cases per year

About how many individuals born with DSD have you seen over your entire career?
________cases over entire career

Please indicate the average number of hours per week that you spend in patient care.
_______hours per week

What is your area of specialization?
- ☐ Urology
- ☐ Endocrinology
- ☐ Other: ______

Please describe the community in which your main office/practice is located.
- ☐ Large metropolitan: (Total city and suburban population: 1,000,000 and over)
- ☐ Small metropolitan: (Total city and suburban population: 50,000 - 999,999)
- ☐ Nonmetropolitan/Rural: (Total population: 49,999 or less)

For US Addresses, please indicate the first digit of your office zip code.
____

Where is your practice located?
- ☐ United States
- ☐ Canada
- ☐ Mexico
- ☐ Other

[Note: the following practice location data were not directly collected in 2003 and 2010, but derived from other items (zip code) + recruitment material and added to the dataset]

[2020: if practice location is United States]
Where is your practice located: State or District (USA)

[2020: if practice location is Canada]
Where is your practice located: Providence or Territory (Canada)

[2020: if practice location is Mexico]
Where is your practice located: State (Mexico)

[2020: if practice location is Other]
Where is your practice located: _______

Primary Practice Setting:
- ☐ Solo or two-physician practice
- ☐ Group practice
- ☐ HMO
- ☐ Medical school or hospital-based
- ☐ Other patient care employment: ______________________________________
- ☐ Other non-patient care employment: ______________________________________[2010 only]

[if (2003 or 2010) AND if practice setting – medical school or hospital]
If you are medical school or hospital-based, please indicate the percentage of time devoted to the following in your medical school or hospital-based practice:

% research ______
% patient care ______
% other ______

What is your gender?
- ☐ Male
- ☐ Female
- ☐ Other, specify:____________________

In what year were you born?

___ ___ ___ ___

CLINICAL CASE PRESENTATIONS

[Note: The option for "other" gender was added in the 2020 survey; previously, there was no option to recommend gender, subsequent items were based on a presumed gender of rearing as a girl) and the remainder of cases were limited to boy vs girl]

Undervirilized 46, XY whose penis when first examined at birth could be palpated only as a thin cord of tissue and with all the suprapubic adipose tissue pushed back, the penis measured 1 cm in length. The urinary meatus terminated at the tip of the penis. Testosterone treatment (25 mg testosterone cypionate once a month for 3 months) increased penis length to 1.8 cm (or –2.5 standard deviations below age-adjusted norms) with substantial increase in diameter. Testes were small (<0.5 cm) and somewhat soft in consistency. At 4 days of life, before testosterone treatment was begun, FSH and LH were significantly elevated for age and testosterone was 15 ng/dL. The diagnosis was considered to be primary testicular failure.

[Note: Two color photographs were shown side-by-side. Each showed external genitalia as described in the text. The first picture (left side panel) presented a front view; the second (right side panel) presented a side view.]

[Note: "/gender of rearing" was added in the 2020 survey]

3a. In your professional judgment, which sex assignment/gender of rearing would result in the best long-term quality of life outcome ['sex assignment' does NOT necessarily imply genital surgery]?

- ☐ Boy
- ☐ Girl
- ☐ Other (e.g., Intersex, non-binary)

[if sex assignment is Boy]

3b. Who should decide whether genital surgery (hypospadias repair) should be performed?

- ☐ The patient should make the decision (likely during adolescence)
- ☐ The parents should make the decision in conjunction with the physician specialists (Endocrinologists and Urologists)

[if sex assignment is Boy AND if who decides is Parents]

3c. **In your professional judgment, genital surgery (neophallus construction) should be performed . . .**

Before 6 months	☐
Before 1 year	☐
Before school entry	☐
During pre-adolescence (Ages 6-10 years)	☐
Adolescence (11 years or older)	☐
I would recommend against surgery	☐

[if sex assignment is Boy]

3d. Surgery is sometimes completed at an early age such that the boy will have no memory of the procedure. If surgery had been performed at such an age in the case of this particular patient, do you think that information regarding the details of the surgery should be disclosed to the patient? If so, when?

Disclosure before school entry (5 years)	☐
Disclosure during middle childhood (6-10 years)	☐
Disclosure during adolescence (11-17 years)	☐
Disclosure during adulthood (18 years or older)	☐
I would recommend against disclosure	☐

[if sex assignment is Girl]

3e. Who should decide whether genital surgery (genitoplasty/vulvaplasty, vaginoplasty) should be performed?

- ☐ The patient should make the decision (likely during adolescence)
- ☐ The parents should make the decision in conjunction with the physician specialists (Endocrinologists and Urologists)

[if sex assignment is Girl AND if who decides is Parents]

3f. In your professional judgment, genital surgery (genitoplasty/vulvaoplasty, vaginoplasty) should be performed…

	Genitoplasty / vulvaplasty	Vaginoplasty
Before 6 months	☐	☐
Before 1 year	☐	☐
Before school entry	☐	☐
During pre-adolescence (Ages 6-10 years)	☐	☐
Adolescence (11 years or older)	☐	☐
I would recommend against surgery	☐	☐

[if sex assignment is Girl]

3g. Genital surgery is sometimes completed at an early age such that the child will have no memory of the procedure. If surgery had been performed at an early age in the case of this particular child, do you think that information regarding details of the surgery or karyotype should be disclosed to the patient?

	Genital surgery	Karyotype
Disclosure before school entry (5 years)	☐	☐
Disclosure during middle childhood (6-10 years)	☐	☐
Disclosure during adolescence (11-17 years)	☐	☐
Disclosure during adulthood (18 years or older)	☐	☐
I would recommend against disclosure	☐	☐

Undervirilized 46, XY male with 1.2 cm phallus with chordee and perineal hypospadias. Basal hormone LH and FSH level at 2 days of life were clearly elevated for age consistent with a lack of an intact feedback system. Testosterone treatment (25 mg testosterone cypionate) resulted in some redness and swelling of the penile skin, with measurement increasing slightly to about 1.7 cm. The child had no suggestion of any other problems. A presumptive diagnosis of partial androgen insensitivity syndrome was assigned.

[Note: One color photograph was presented showing the external genitalia as described in the text. Tissue surrounding the phallus is shown retracted between a gloved finger and thumb; a measuring tape is present.]

[Note: "/gender of rearing" was added in the 2020 survey; previously, it read "… which sex assignment would result in…"]

4a. In your professional judgment, which sex assignment/gender of rearing would result in the best long-term quality of life outcome ['sex assignment' does NOT necessarily imply genital surgery]?

- ☐ Boy
- ☐ Girl
- ☐ Other (e.g., Intersex, non-binary)

[if sex assignment is Boy]

4b. Who should decide whether genital surgery (hypospadias repair, neophallus construction) should be performed?

- ☐ The patient should make the decision (likely during adolescence)
- ☐ The parents should make the decision in conjunction with the physician specialists (Endocrinologists and Urologists)

[if sex assignment is Boy AND if who decides is Parents]

4c. In your professional judgment, genital surgery (hypospadias repair, neophallus construction) should be performed . . .

	Hypospadias repair	Neophallus construction
Before 6 months	☐	☐
Before 1 year	☐	☐
Before school entry	☐	☐
During pre-adolescence (Ages 6-10 years)	☐	☐
Adolescence (11 years or older)	☐	☐
I would recommend against surgery	☐	☐

[if sex assignment is Boy]

4d. Surgery is sometimes completed at an early age such that the boy will have no memory of the procedure. If surgery had been performed at such an age in the case of this particular patient, do you think that information regarding the details of the surgery should be disclosed to the patient? If so, when?

Disclosure before school entry (5 years)	☐
Disclosure during middle childhood (6-10 years)	☐
Disclosure during adolescence (11-17 years)	☐
Disclosure during adulthood (18 years or older)	☐
I would recommend against disclosure	☐

[if sex assignment is Girl]

4e. Who should decide whether genital surgery (genitoplasty/vulvaplasty, vaginoplasty) should be performed?

- ☐ The patient should make the decision (likely during adolescence)
- ☐ The parents should make the decision in conjunction with the physician specialists (Endocrinologists and Urologists)

[if sex assignment is Girl AND if who decides is Parents]

4f. In your professional judgment, genital surgery (genitoplasty/vulvaoplasty, vaginoplasty) should be performed

	Genitoplasty / vulvaplasty	Vaginoplasty
Before 6 months	☐	☐
Before 1 year	☐	☐
Before school entry	☐	☐
During pre-adolescence (Ages 6-10 years)	☐	☐
Adolescence (11 years or older)	☐	☐
I would recommend against surgery	☐	☐

[if sex assignment is Girl]

4g. Genital surgery is sometimes completed at an early age such that the child will have no memory of the procedure. If surgery had been performed at an early age in the case of this particular child, do you think that information regarding details of the surgery or karyotype should be disclosed to the patient?

	Genital surgery	Karyotype
Disclosure before school entry (5 years)	☐	☐
Disclosure during middle childhood (6-10 years)	☐	☐
Disclosure during adolescence (11-17 years)	☐	☐
Disclosure during adulthood (18 years or older)	☐	☐
I would recommend against disclosure	☐	☐

46, XY. Mishandled circumcision within the first week of life resulting in complete penile ablation. Testes were of normal size and consistency, and fully descended. LH, FSH, and testosterone were normal for a male child at 6 days of age.

[Note: Only a written description was presented; no photographs]

[Note: "/gender of rearing" was added in the 2020 survey]

5a. In your professional judgment, which sex assignment/gender of rearing would result in the best long-term quality of life outcome ['sex assignment' does NOT necessarily imply genital surgery]?

- ☐ Boy
- ☐ Girl
- ☐ Other (e.g., Intersex, non-binary)

[if sex assignment is Boy]

5b. Who should decide whether genital surgery (hypospadias repair) should be performed?

- ☐ The patient should make the decision (likely during adolescence)
- ☐ The parents should make the decision in conjunction with the physician specialists (Endocrinologists and Urologists)

[if sex assignment is Boy AND if who decides is Parents]

5c. In your professional judgment, genital surgery (eg. neophallus construction using a forearm free flap) should be performed . . .

- Before 6 months ☐
- Before 1 year ☐
- Before school entry ☐
- During pre-adolescence (Ages 6-10 years) ☐
- Adolescence (11 years or older) ☐
- I would recommend against surgery ☐

[if sex assignment is Boy]

5d. Surgery is sometimes completed at an early age such that the boy will have no memory of the procedure. If surgery had been performed at such an age in the case of this particular patient, do you think that information regarding the details of the surgery should be disclosed to the patient? If so, when?

- Disclosure before school entry (5 years) ☐
- Disclosure during middle childhood (6-10 years) ☐
- Disclosure during adolescence (11-17 years) ☐
- Disclosure during adulthood (18 years or older) ☐
- I would recommend against disclosure ☐

[if sex assignment is Girl]

5e. Who should decide whether genital surgery (genitoplasty/vulvaplasty, vaginoplasty) should be performed?

- ☐ The patient should make the decision (likely during adolescence)
- ☐ The parents should make the decision in conjunction with the physician specialists (Endocrinologists and Urologists)

[if sex assignment is Girl AND if who decides is Parents]

5f. In your professional judgment, genital surgery (genitoplasty/vulvaoplasty, vaginoplasty) should be performed

	Genitoplasty / vulvaplasty	Vaginoplasty
Before 6 months	☐	☐
Before 1 year	☐	☐
Before school entry	☐	☐
During pre-adolescence (Ages 6-10 years)	☐	☐
Adolescence (11 years or older)	☐	☐
I would recommend against surgery	☐	☐

[if sex assignment is Girl]

5g. Genital surgery is sometimes completed at an early age such that the child will have no memory of the procedure. If surgery had been performed at an early age in the case of this particular child, do you think that information regarding details of the surgery or karyotype should be disclosed to the patient?

	Genital surgery	Karyotype
Disclosure before school entry (5 years)	☐	☐
Disclosure during middle childhood (6-10 years)	☐	☐
Disclosure during adolescence (11-17 years)	☐	☐
Disclosure during adulthood (18 years or older)	☐	☐
I would recommend against disclosure	☐	☐

Participants

Recruitment - Procedures
At each timepoint, the research team sought approval from leadership of both the (Lawson Wilkins) Pediatric Endocrine Society (PES) the Societies for Pediatric Urology (SPU) to survey their membership and to provide member rosters that include contact information. Leadership of each society approved the research provided rosters, apart from PES at T3 2020 - citing concerns about burden. As such, at T3 for PES, only those who had previously been invited to participate at T1 or T2 were invited. A publicly available directory PES was reviewed to remove names of those who were no longer listed as PES members; no new members were added.

To account for slightly differing timelines for study approval by PES and SPU and to manage the number of individuals targeted for participation and anticipated follow-up reminders, survey invitations were sent in waves, rather than to all participants at once. Invitation letters that included an explanation of the study and survey login instructions were sent society members in 2003-04 (T1), 2010-11 (T2), and 2020 (T3). Participants were also offered a paper-and-pencil version upon request. To optimize recruitment, eligible respondents received up to three follow-up requests to participate. After rates of survey completion dropped to minimal levels for several weeks, final requests for participation to non-responders took the form of a phone call and/or a single-page faxed letter encouraging either participation or to otherwise provide a reason for declining to participate at T1. Through this process, it was learned non-responders were frequently either retired, not in clinical practice, were exclusively involved in research, or not providing care to patients with a DSD. At T2 and T3, final follow-ups took place via email. Additionally, a screening and opt-out survey was added to the beginning of the T3 survey to simplify and streamline efforts at identifying participant (in)eligibility and reduce burden associated with follow-ups contacts for non-responders.

Eligibility
Common across all timepoints: Current members of either PES or SPU

Table 3. Eligibility requirements unique to specific timepoint:

		T1	T2	T3
Clinical Management	Is currently providing care for patients with intersex/DSD conditions	X	X	X
	Has not provided care for patients with intersex/DSD conditions, but are in a position where they could, potentially, do so now or in the future or otherwise influence clinical care	X		
	Provided care for patients with intersex/DSD conditions in past only[1]	X	X	X
Professional Background	Pediatric Endocrinologist, Other Endocrinologist	X	X	X
	Pediatric Urologist, Other Urologist	X	X	X
	Other professional degree (eg, PhD)	X		
Practice Location	United States	X	X	X
	Canada	X	X	X
	Mexico	X	X	X
	Other		X	

[1] Emeriti were, initially, included in the T1 survey; a combination of difficulty reaching these potential participants due to missing or inaccurate contact information, low participation rates, and feedback from several who targeted for participation that they are retired / unable to provide valid input (in their estimation) lead to discontinuing recruitment of emeriti as the project progressed.

Participants were promised confidentially of their responses; procedures were approved by the authors' Institutional Review Boards at the University at Buffalo School of Medicine and Biomedical Sciences (T1 2003) and University of Michigan Medical School (T2 2010 and T3 2020).

Participation Rates and Demographics

Given small differences in eligibility criteria for recruitment, participation rates are calculated using the most restrictive eligibility criteria.

Table 4. Participant ascertainment, recruitment, and participation

	T1 2003		T2 2010		T3 2020	
	PES	SPU	PES	SPU	PES	SPU
Names in Directory	764	263	868	237	494[6]	354
Ineligible[1]						
Co-I, FG, or PT participant[2]	14	9	12	8	10	8
Emeriti	117					
Retired	11	5	10	6	10	21
Deceased	2		1			
No DSD patients	34	6	29	3	15	37
No clinical practice	16	1	16		4	3
Practice outside N. America	1		13		8	2
Other; >1 criteria met	53	52	10	2	13	2
Eligible, invited, sample	516	190	777	218	434	281
Participated	297 (57.6%)	132 (69.5%)	319 (41.1%)	116 (53.2%)	114 (26.3%)	150 (53.4%)
Skipped all 46,XY items[3]	3	0	4	2	4	4
Logged in / consented only[4]	--	--	--	--	5	10
Declined participation[5]	48	10	27	3	5	5
No Response	168	48	425	96	304	107
Eligible but not invited	0	0	1	1	0	0
No contact information	0	0	1	0	2	5

[1] Ineligibility was determined at multiple stages. Determinations were made prior to sending survey invitations to members; for others, it occurred after invitations were sent. It is possible that some of those for whom no responses are recorded are ineligible.

[2] Co-investigators, focus group members, and pilot test participants involved in the design of this project.

[3] Participants were free to skip over questions they did not wish to answer and discontinue participation at any time; some completed other portions of the survey without completing any items related to the 46XY cases

[4] At T3, several targeted participants had logged into the survey and completed portions of the screening survey or demographics, but did not provide responses to items in any other section; this was not possible in earlier years.

[5] A common reason cited for declining participation was being "too busy."

[6] Reflects an error in recruitment

[7] Only current members of PES who had participated in the past were included in the 2020 PES sample; this does not represent the total number of names listed in the PES directory.

Provided they maintained membership in their respective professional society over time, members had the opportunity to participate in up to three waves of the survey. The majority participated on only one occasion; however, within-subjects comparisons are made possible on a limited basis:

Table 5. Participation over time: 46,XY clinical case presentation items

Participation	Participants (n)		
	PES	SPU	Total (PES+SPU)
T1 only	151	51	202
T2 only	131	32	163
T3 only	1	92	93
T1 & T2	87	36	123
T1 & T3	12	10	22
T2 & T3	54	13	67
T1, T2, & T3	47	35	82

Survey Completeness

In line with research ethics, participants were free to skip over questions they did not wish to answer and discontinue participation at any time. As such, not all surveys have 100% of items answered – the use of branching and skip logic notwithstanding.

Given the order in which major sections of the survey were presented and order of items within sections (see Survey Components, above), discontinuation disproportionately affected response rates of individual items depending on the year of administration.

Table 6. Participant demographics: 46,XY clinical case presentation items

	T1 2003				T2 2010				T3 2020			
	PES		SPU		PES		SPU		PES		SPU	
	n	%	n	%	n	%	n	%	n	%	n	%
Sex												
• Male	180	60.6	126	95.5	159	49.8	109	94.0	58	50.9	126	84.0
• Female	117	39.4	6	4.5	160	50.2	7	6.0	55	48.2	24	16.0
• Other[1]	--	--	--	--	--	--	--	--	1	0.9	0	0
Practice Community[2]												
• Large Metropolitan	173	60.7	86	71.7	199	62.4	80	69.0	79	69.3	112	74.7
• Small Metropolitan	107	37.5	33	27.5	120	37.6	34	29.3	34	29.8	36	24.0
• Nonmetropolitan / Rural	5	1.8	1	0.8			2	1.7	1	0.9	2	1.3
Practice Country												
• United States	279	93.9	125	94.7	301	94.4	111	95.7	106	93.0	143	95.3
• Canada	18	6.1	7	5.3	18	5.6	5	4.3	8	7.0	7	4.7
Practice Setting[3]												
• Medical School or Hospital	212	76.8	78	65.5	247	77.4	72	62.1	96	84.2	109	72.7
• Solo or 2-physician Practice	22	8.0	16	13.4	72	22.6	44	37.9	5	4.4	7	4.7
• Group Practice	33	12.0	24	20.2					11	9.6	31	20.7
• HMO	9	3.3	1	0.8					1	0.9	3	2.0
• Other	0	0	0	0					1	0.9	0	0
	mean	SD	mean	SD	mean	SD	mean	SD	mean	SD	mean	SD
Year of Birth	1950.9	9.1	1951.5	8.9	1960.0	10.9	1955.7	8.2	1960.7	9.2	1966.1	9.4
Cases Seen												
• In Past Year	10.1	25.4	8.3	8.6	14.8	57.7	11.1	16.3	13.4	20.4	11.3	18.2
• Over Career	65.9	87.8	91.3	121.8	84.4	135.2	133.3	232.5	99.3	154.6	135.2	432.0
Hours/Week in Patient Care	28.0	15.6	52.6	15.5	28.5	15.7	49.8	15.4	26.2	12.7	46.0	13.4
Proportion of Time Spent in[4]												
• Research	25.0	23.4	11.5	11.0	27.7	27.4	11.0	9.9	--	--	--	--
• Patient Care	55.8	25.5	77.6	13.8	56.4	27.2	77.5	15.4	--	--	--	--
• Other	19.2	16.6	10.9	11.2	16.2	14.7	11.5	12.6	--	--	--	--

Note: Not all participants answered every item; percentages are calculated based on number of valid responses to each item rather than on cohort size
[1] "Other" was included as a response option at T3 only; [2] Small Metropolitan and Nonmetropolitan / Rural were collapsed for PES members at T2; [3] Practice Setting was parsed into 2 categories at T2; [4] Items was asked only of those who indicated working in a "Medical School of Hospital" Practice setting; Item was not included at T3
